# Unravelling heterogeneous malaria transmission dynamics in the Peruvian Amazon: insights from a cross-sectional survey

**DOI:** 10.1186/s12936-024-05032-8

**Published:** 2024-07-15

**Authors:** Viviana Pinedo-Cancino, Katty M. Arista, G. Christian Baldeviano, Rafael Saavedra-Langer, Andrei Arana, Maria E. Vásquez-Chasnamote, Andree Valle-Campos, Juan C. Castro, Julio A. Ventocilla, Edward S. Smith, Andres G. Lescano, Lastenia Ruíz-Mesia

**Affiliations:** 1 Laboratorio de Investigación de Productos Naturales Antiparasitarios de la Amazonía (LIPNAA), Centro de Investigaciones de Recursos Naturales de la UNAP (CIRNA), Universidad Nacional de la Amazonía Peruana (UNAP), Iquitos, Peru; 2 Facultad de Medicina Humana, Universidad Nacional de la Amazonía Peruana (UNAP), Iquitos, Peru; 3 U.S. Naval Medical Research Unit SOUTH (NAMRU SOUTH), Bellavista, Callao Peru; 4 Departamento de Bioquímica E Inmunología, Instituto de Ciencias Biológicas, Universidad Federal de Minas Gerais, Belo Horizonte, Minas Gerais, Brazil; 5 Departamento de Ciencias Biológicas, Universidad Nacional Mayor de San Marcos (UNMSM), Lima, Peru; 6 Unidad Especializada del Laboratorio de Investigación en Biotecnología, Centro de Investigaciones de Recursos Naturales de la UNAP (CIRNA), Universidad Nacional de la Amazonía Peruana (UNAP), Iquitos, Peru; 7 Departamento Académico de Ciencias Biomédicas y Biotecnología, Facultad de Ciencias Biológicas, Universidad Nacional de la Amazonía Peruana (UNAP), Iquitos, Peru; 8 Vysnova Partners Inc., Rockville, MD USA; 9 Facultad de Medicina Humana, Universidad Peruana Cayetano Heredia (UPCH), Lima, Peru; 10 Facultad de Ingeniería Química, Universidad Nacional de la Amazonía Peruana (UNAP), Iquitos, Peru; 11 Present Address: School of Medicine, Universidad Continental, Huancayo, Peru; 12 Present Address: Facultad de Medicina Humana, Universidad Peruana Cayetano Heredia (UPCH), Lima, Peru; 13 Present Address: Bluebird Bio, Inc., Somerville, MA USA; 14 Present Address: Clima, Latin American Center of Excellence for Climate Change and Health, and Emerge, Emerging Diseases and Climate Change Research Unit, School of Public Health and Administration, Universidad Peruana Cayetano Heredia (UPCH), Lima, Peru

**Keywords:** Asymptomatic infections, Seroepidemiologic studies, Malaria prevalence, Public health, Risk factors, Seroconversion

## Abstract

**Background:**

Malaria remains a global health challenge, particularly in Peru's Loreto region. Despite ongoing efforts, high infection rates and asymptomatic cases perpetuate transmission. The Peruvian Ministry of Health’s “Zero Malaria Plan” targets elimination. This novel study combines microscopic, molecular, and serological techniques to assess transmission intensity, identify epidemiological risk factors, and characterize species-specific patterns across villages. The findings aim to inform targeted interventions and support broader malaria elimination efforts in line with the Zero Malaria Plan initiative.

**Methods:**

A cross-sectional malaria survey was conducted in the Zungarococha community, comprising the villages Llanchama (LL), Ninarumi (NI), Puerto Almendra (PA), and Zungarococha (ZG), using microscopic, molecular, and serological techniques to evaluate malaria transmission intensity. Statistical analysis, including multivariate-adjusted analysis, seroprevalence curves, and spatial clustering analysis, were performed to assess malaria prevalence, exposure, and risk factors.

**Results:**

The survey revealed a high prevalence of asymptomatic infections (6% by microscopy and 18% by PCR), indicating that molecular methods are more sensitive for detecting asymptomatic infections. Seroprevalence varied significantly between villages, reflecting the heterogeneous malaria transmission dynamics. Multivariate analysis identified age, village, and limited bed net use as significant risk factors for malaria infection and species-specific exposure. Seroprevalence curves demonstrated community-specific patterns, with Llanchama and Puerto Almendra showing the highest seroconversion rates for both *Plasmodium* species.

**Conclusions:**

The study highlights the diverse nature of malaria transmission in the Loreto region, particularly nothing the pronounced heterogeneity as transmission rates decline, especially in residual malaria scenarios. The use of molecular and serological techniques enhances the detection of current infections and past exposure, aiding in the identification of epidemiological risk factors. These findings underscore the importance of using molecular and serological tools to characterize malaria transmission patterns in low-endemic areas, which is crucial for planning and implementing targeted interventions and elimination strategies. This is particularly relevant for initiatives like the Zero Malaria Plan in the Peruvian Amazon.

**Supplementary Information:**

The online version contains supplementary material available at 10.1186/s12936-024-05032-8.

## Background

Malaria remains a significant global health challenge, causing substantial morbidity and mortality, particularly in tropical and subtropical regions. According to the 2022 World Health Organization [1], malaria is a major socioeconomic and health problem that affects half of the world's population. In the Americas alone, an estimated 20 million people are at high risk of contracting this debilitating disease.

Peru's Loreto region, located in the Amazon basin, has historically experienced the highest malaria burden in the country [2]. Between 1995 and 1998, epidemics struck the region's capital city, Iquitos, following the abandonment of fumigation programmes in the 1980s [3] Despite ongoing efforts, the situation remains critical, with approximately 59,000 cases reported in 2015 alone [4]. Exacerbating this problem, the region is characterized by a large proportion of asymptomatic cases [2, 3], which can perpetuate transmission.

These epidemic outbreaks and the overall increase in malaria transmission, particularly in rural and remote areas, have far-reaching consequences. They not only affect the general population, such as loggers and farmers, but also pose a significant risk to deployed U.S. armed forces operating in the region.

In response to this dire situation, the Peruvian Ministry of Health (MINSA) by Resolution No. 244-2017, approved the technical document "Zero Malaria Plan period 2017–2021" proposed by the General Direction of Strategic Interventions in Public Health with the general objective of developing a programme to eliminate this disease in the Amazon region with a community and intercultural approach (http://www.minsa.gob.pe/?op=51&note=23877). However, the success of such initiatives relies heavily on the availability of reliable diagnostic tools capable of monitoring temporary changes in the transmission intensity and guiding the development of effective interventions.

Although previous studies have investigated malaria in the Peruvian Amazon, this study presents a novel approach that combines microscopic, molecular, and serological techniques in a comprehensive cross-sectional survey. The integration of these diagnostic methods allows for a more robust assessment of both current infections and historical exposure, enabling a deeper understanding of the heterogeneous transmission dynamics. Moreover, spatial clustering analysis and seroconversion modeling provide valuable insights into the varying transmission patterns across different villages within a localized area. This multifaceted approach contribute to the development of targeted interventions and elimination strategies in regions with residual malaria transmission.

Technological advancements in seroepidemiological studies have enhanced their effectiveness as tools for assessing malaria transmission, particularly in areas where parasite prevalence is low [2, 5–8]. Nonetheless, there is a need to standardize ELISA protocols [9], especially in endemic regions characterized by unstable and heterogeneous transmission, such as the Peruvian Amazon [2, 10]. Therefore, the main objective of this study was to unravel the heterogeneous dynamics of malaria transmission in the Peruvian Amazon, particularly in the Loreto region, by conducting a comprehensive cross-sectional survey using a combination of microscopic, molecular, and serological techniques.

Furthermore, this study aimed to evaluate the malaria transmission intensity, identify epidemiological risk factors, and characterize species-specific transmission patterns across different villages within the study area. The findings can inform targeted interventions and support the broader goal of malaria elimination in the region, aligning with the objectives of the Zero Malaria Plan initiative.

## Methods

### Study site and population

**Fig. 1:**
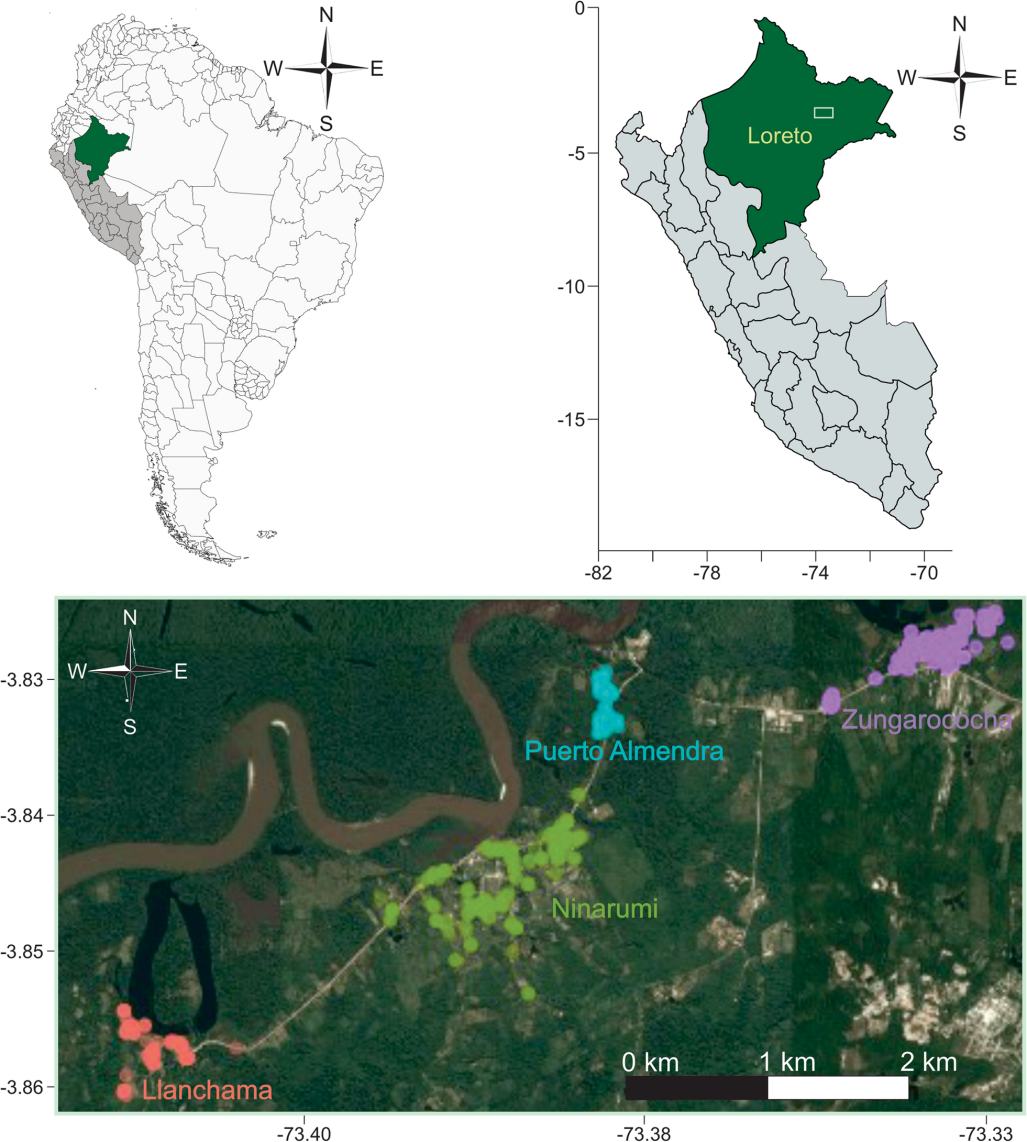
Geographical localization of the four villages of the community of Zungarococha, Loreto, Peru

The study population comprised the Malaria Immunology and Genetics in the Amazon (MIGIA) cohort, which investigated approximately 2000 individuals residing in the community of Zungarococha (Fig. 1), located in the San Juan District, south of Iquitos, Peru. In this region, *Plasmodium falciparum* and* Plasmodium vivax* infections are frequently asymptomatic, characterized by low parasite density [3, 11]. The community of Zungarococha consists of four villages: Llanchama (LL), Ninarumi (NR), Puerto Almendra (PA), and Zungarococha (ZG). These villages are located approximately two kilometers apart and are served by the same health post operated by MINSA. As described in more detail by Branch et al*.* [3], the environmental conditions and socioeconomic levels across these villages are similar.

A cross-sectional survey was conducted between July and August 2015. During this period, the study team visited each household in the Zungarococha villages and invited local residents to participate in the seroepidemiological study.

After obtaining informed consent or assent from the participants, examinations were conducted to assess fever and malaria symptoms. Subsequently, a finger-prick blood sample was collected in a microtube containing the anticoagulant EDTA. Slides of blood smears (for microscopic diagnosis), and plasma and red blood cells were obtained for serological and molecular diagnosis, respectively. All samples were transported to the CIRNA (Centro de Investigaciones de Recursos Naturales de la UNAP) in Iquitos for further analysis.

All participants with a positive malaria diagnosis by microscopy were promptly treated at MINSA-affiliated hospitals or health centres following the National Drug Policy Guidelines of the Peruvian Ministry of Health.

### Microscopic diagnosis

Microscopy was performed at the CIRNA by two experienced microscopists following standard procedures as previously described by Branch et al. [3]. Blood smears were stained with 10% Giemsa stain using standard protocols. Trophozoites and gametocytes of all malaria species were counted separately under 100× magnification, using oil immersion. Negative microscopy results were confirmed if no *Plasmodium* parasites were found after examining at least 500 white blood cells.

For species confirmation and quality control, all blood smears positive for malaria and at least 20% of those negative for malaria were sent to the U.S. Naval Medical Research Unit SOUTH (NAMRU SOUTH).

### Molecular diagnosis

DNA was isolated from blood samples using a DNeasy Blood and Tissue Kit (Qiagen Inc., Valencia, CA, USA) as previously described [12]. Additionally, the semi-nested multiplex malaria PCR molecular method was employed for detecting *Plasmodium* species [13]. This method involves a two-tube multiplex PCR with an initial genus-specific amplification followed by secondary amplification using a universal *Plasmodium* primer and species-specific reverse primers. The targeted gene region for amplification in this method is the small subunit ribosomal RNA (SSU rRNA) gene of *Plasmodium*. This approach enables the detection and differentiation of four human malarial species in blood samples. Semi-nested multiplex PCR is widely recognized as a molecular gold standard due to its excellent performance in detecting mixed-species infections and its capability to distinguish the four species of *Plasmodium*.

### Serologic diagnosis

The ELISA for total IgG detection was conducted according to previously established protocols [14, 15]. Briefly, 50 µl/well of the recombinant 19-kDa conserved C-terminal part of the merozoite surface protein 1 of *P. falciparum* (PfMSP1-19) or *P. vivax* (PvMSP1-19) at concentrations of 0.5 µg/ml and 0.2 µg/ml, respectively, in borate-buffered saline, were coated onto the plates and incubated overnight at 4 °C. Subsequently, the plates were washed with phosphate-buffered saline containing 0.05% Tween 20 and blocked with 1% Bovine Serum Albumin (BSA) in borate-buffered saline.

Sera samples were diluted (1:200) in AB buffer (0.15 M Na_2_HPO_4_, 0.15 M NaH_2_PO4, NaCl, 0.05% Tween 20, and 0.05% BSA) containing 1.5% nonfat skimmed milk, and added in duplicate and incubated at room temperature for 1–2 h. After washing three times with AB buffer, the bound antibodies were detected using peroxidase-conjugated goat-anti-human-IgG (Chemicon, Billerica, MA, USA) diluted 1:6000 for 1 h. The wells were subsequently washed thrice with AB buffer. The substrate 3,3′,5,5′-tetramethylbenzidine (KPL, Gaithersburg, MD, USA) was added to each well and incubated in the dark at room temperature for 30 min. The reaction was stopped by adding 25 µl of 0.25 M HCL, and the absorbance at 450 nm was measured using an ELISA plate reader (DYNEX, Chantilly, VA, USA).

To ensure quality control, ten serum samples from healthy, unexposed individuals from Peru and the United States served as negative controls. A "positive pool" control, composed of samples from individuals infected with *P. falciparum* or *P. vivax*, was included in each plate, with dilutions ranging from 1/50 to 1/102400, following the methodology described by Clark et al. [11].

### Statistical analysis

Data were entered into Excel (Microsoft Corp., USA), and the survey and prevalence data were analyzed using the statistical software R version 3.4.2 [16]. The hypothesis of differences in proportions across villages was tested using the Pearson Chi-square test for categorical variables in both datasets.

For ELISA results, inter-plate standardization using serially diluted reference serum (from 1/50 to 1/102400) was performed on each plate using dose–response analysis [17]. Antibody units (AU) were estimated from OD replicates per sample using a symmetric four-parameter log-logistic model [18]. Seropositivity was classified by applying a mixture model to the AU distribution [19]. The optimal model was determined based on two criteria: the Akaike Information Criterion (AIC) and the proportion of unclear classifications. The model with the lowest AIC value and the lowest rate of unclear classifications was selected as the best fit. A minimum classification probability of 90% was set as a threshold to ensure reliable assignments.

Multivariate-adjusted analysis using logistic regression models was applied to determine the association between the survey covariates and malaria infection (positive PCR) or species-specific exposure (seropositivity) as dichotomous outcome variables. Specifically, a binomial family generalized linear model with a logarithmic link function was used to estimate odds ratios (ORs) for the individual-level variables. AIC was used as a stopping rule to perform backward elimination of these variables, thereby identifying the best-fitting model.

To estimate the serological conversion and reversion rates for each village and species, a reversible catalytic model was fitted using age-stratified seroprevalence data in decile groups. The seroconversion rate (SCR) and seroreversion rate (SRR) were defined according to Bekessy et al. [20] and Charlwood et al. [21]. These rates represent the changes in individuals from seronegative to seropositive and vice versa, respectively. This model assumes a constant and stable malaria transmission intensity over time, with no migration and no differences in SCR among individuals within the same community.

The local Moran’s I test was used to detect spatial clusters [22, 23]. The unit of analysis was the proportion of individuals who tested positive for infection per household, as determined by both microscopy and PCR for any species. QGIS [24] was used to re-project the raw coordinates, and GeoDa [25] was utilized to perform the clustering analysis. Given the uneven distance between households, spatial weighting was conducted using ArcGIS [26].

### Ethical aspects

The study protocol (NAMRU6.2014.0031) was approved by the Institutional Review Board of NAMRU SOUTH, in compliance with all applicable federal regulations governing the protection of human subjects. Permissions were obtained from the health and local authorities after the purpose and procedures of the survey were explained. All individuals enrolled in this study provided signed informed consent or assent.

## Results

A total of 936 individuals were recruited from the community of Zungarococha, comprising 116 from LL, 286 from NR, 106 from PA, and 428 from ZG (Table 1). Although females made up 55% of the sample (513/936), this difference was not statistically significant across villages (*P* = 0.187). The age distribution showed that half of the participans were under 18 years old, with a consistent age stratification pattern across villages (*P* = 0.205). Among participants aged 18 and older, 46% had completed primary education, and 27% had completed secondary education. The most common occupation reported by participants aged 15 and older was the housewife (60%). The community had a significant proportion of individuals engaged in outdoor activities, such as farming, guarding, logging, and fishing (17%), as well as indoor activities, including laboring, trading, and driving (10%). Notably, 84% of participants reported using bed nets for sleeping, although only 48% reported the presence of insecticides. Furthermore, 92% of participants had resided in the area for more than two years, indicating a relatively stable population with few recent immigrants. Finally, 45% of the population self-reported never having experienced a malaria episode in the past.

**Table 1:** Baseline characteristics of enrolled individuals during a cross-sectional study in the four villages of the community of Zungarococha, Loreto, Peru

Characteristics	N	Llanchama	Ninarumi	Puerto Almendra	Zungarococha	Combined	*P*-value
		*N* = 116	*N* = 286	*N* = 106	*N* = 428	*N* = 936	
		% (n)	% (n)	% (n)	% (n)	% (n)	
*Sex*							
Male	936	49 (57)	42 (119)	40 (42)	48 (205)	45 (423)	0.187
Female		51 (59)	58 (167)	60 (64)	52 (223)	55 (513)	
*Age groups (years)*							
≤ 8	936	31 (36)	28 (81)	26 (28)	21 (91)	25 (236)	0.205
9–17		23 (27)	28 (81)	22 (23)	26 (112)	26 (243)	
18–36		24 (28)	19 (55)	27 (29)	26 (113)	24 (225)	
> 36		22 (25)	24 (69)	25 (26)	26 (112)	25 (232)	
*Education (≥ 18 years old)*							
Primary	455	38 (20)	52 (64)	60 (33)	41 (93)	46 (210)	0.012
Incomplete secondary		25 (13)	24 (30)	20 (11)	22 (49)	23 (103	
Complete secondary		33 (17)	16 (20)	18 (10)	34 (76)	27 (123)	
None		4 (2)	7 (9)	2 (1)	3 (7)	4 (19)	
*Occupation (≥ 15 years old)*							
Farmer/Guard/Logger/Fisher	462	35 (20)	20 (24)	7 (4)	13 (30)	17 (78)	< 0.001
Housewife		54 (31)	63 (76)	63 (34)	58 (134)	60 (275)	
Laborer/Trader/Driver		2 (1)	6 (7)	13 (7)	13 (29)	10 (44)	
None (Students/Pensioner)		9 (5)	11 (13)	17 (9)	16 (38)	14 (65)	
*Electricity availability*							
Yes	936	46 (53)	94 (268)	85 (90)	99 (422)	89 (833)	< 0.001
No		54 (63)	6 (18)	15 (16)	1 (6)	11 (103)	
*Predominant material in wall*							
Wood	936	99 (115)	71 (204)	82 (87)	26 (110)	55 (516)	< 0.001
Concrete		0 (0)	26 (73)	16 (17)	73 (313)	43 (403)	
Triplay		1 (1)	3 (9)	2 (2)	1 (5)	2 (17)	
*Predominant material in floor*							
Wood	936	23 (27)	6 (16)	0 (0)	1 (3)	5 (46)	< 0.001
Concrete		8 (9)	11 (31)	26 (28)	79 (336)	43 (404)	
Soil or sand		69 (80)	84 (239)	74 (78)	21 (89)	52 (486)	
*Sleeping under bed net (last 2 weeks)*							
Yes	932	94 (109)	84 (239)	76 (81)	84 (355)	84 (784)	0.004
No		6 (7)	16 (47)	24 (25)	16 (69)	16 (148)	
*Impregnated bed net*							
Yes	784	56 (61)	31 (75)	11 (9)	52 (186)	42 (331)	< 0.001
No		44 (48)	69 (164)	89 (72)	48 (169)	58 (453)	
*Time in village (years)*							
≤ 2	936	4 (5)	13 (37)	9 (10)	6 (25)	8 (77)	0.003
> 2		96 (111)	87 (249)	91 (96)	94 (403)	92 (859)	
*Self-report of confirmed malaria episodes*							
0	931	2 (2)	57 (161)	27 (29)	53 (225)	45 (417)	< 0.001
1		11 (13)	10 (28)	3 (3)	15 (64)	12 (108)	
2–3		56 (65)	20 (56)	45 (48)	18 (77)	26 (246)	
≥ 4		31 (36)	14 (39)	25 (26)	14 (59)	17 (160)	

*N* is the number of non-missing values

Test used: Pearson’s Chi-squared Test for differences between villages

Regarding variables that were significantly different between villages (*P* < 0.05), PA had the highest proportion of individuals with only primary education (60%) and the lowest proportion of outdoor occupation workers (7%). This village also had the lowest proportion of individuals who slept under a bed net in the last two weeks (24%) and the highest proportion of bed net users without impregnation (89%).

Differences in household materials were observed (Table 1). ZG had the highest proportion of houses made of concrete in both structures (97%, n = 296; 73% walls and 79% floors). Conversely, NR had the highest proportion of houses with wood walls and soil/sand floors (42%, n = 160; 71% walls and 84% floors) or concrete walls and soil/sand floor (72%, n = 70; 26% walls). LL had the highest proportion of houses with wood walls and floors (60%, n = 27; 99% walls and 23% floors). Additionally, LL had the lowest proportion of households with electricity (46%) compared to the other villages. Finally, the population from LL had the highest proportion of self-reported malaria episodes (98% at least once), whereas NR and ZG reported that more than 53% of their populations had never experienced a malaria episode (Table 1).

The total parasite prevalence by microscopy was 6%, comprising 44 *Plasmodium vivax* and 10 *Plasmodium falciparum* infections. This prevalence was significantly different across villages for *P. vivax* (*P* = 0.004) and total infections (*P* = 0.02), but not for *P. falciparum* (*P* = 0.41), with each village having a proportion of total infections lower than 2% (Table 2; Additional file 1: Fig. S1). The overall prevalence by PCR was 18%, three times higher than that detected by microscopy, including 154 *P. vivax,* 6 *P. falciparum,* and 6 mixed infections. This prevalence was also significantly different across villages for *P. vivax,* mixed, and total infections (*P* < 0.001), but not for *P. falciparum* (*P* = 0.85). According to PCR results, LL had the highest prevalence of *P. vivax* (27%), followed by PA (25%), NR (18%), and ZG (11%) (Table 2; Additional file 1: Fig. S1).

**Table 2:** Malaria prevalence and seroprevalence in the four villages of the community of Zungarococha, Loreto, Peru

Method of diagnosis	Llanchama	Ninarumi	Puerto Almendra	Zungarococha	Combined	*P*-value
	*N* = 116	*N* = 286	*N* = 106	*N* = 428	*N* = 936	
	% (n)	% (n)	% (n)	% (n)	% (n)	
*Microscopic*						
*P. falciparum*	1 (1)	1 (2)	0 (0)	2 (7)	1 (10)	0.413
*P. vivax*	11 (13)	4 (11)	6 (6)	3 (14)	5 (44)	0.004
Total	12 (14)	5 (13)	6 (6)	5 (21)	6 (54)	0.02
*Molecular*						
*P. falciparum*	1 (1)	1 (2)	0 (0)	1 (3)	1 (6)	0.847
*P. vivax*	27 (31)	18 (52)	25 (26)	11 (45)	16 (154)	< 0.001
Mixed infection	3 (4)	1 (2)	0 (0)	0 (0)	1 (6)	< 0.001
Total	31 (36)	20 (56)	25 (26)	11 (48)	18 (166)	< 0.001
*Serologic*						
*P. falciparum* (MSP1-19 kDa)	22 (26)	13 (36)	15 (16)	5 (21)	11 (99)	< 0.001
*P. vivax* (MSP1-19 kDa)	19 (22)	13 (36)	26 (28)	20 (87)	18 (173)	0.007
Mixed infection	8 (9)	9 (27)	16 (17)	2 (9)	7 (62)	< 0.001
Total	49 (57)	35 (99)	58 (61)	27 (117)	36 (334)	< 0.001

*N* is the number of non-missing values

Test used: Pearson’s Chi-squared Test for differences between villages

In contrast to parasite prevalence, seroprevalence for each community was significantly different for both species (*P* < 0.001). The species-specific seroprevalence showed similar heterogeneity between villages but in a different order (Table 2, Figure S1). Overall, seropositivity in PA was high (26% for *P. vivax* MSP1-19 and 15% for *P. falciparum* MSP1-19) and relatively high in LL (19% and 22%) and NR (13% and 13%) for *P. vivax* MSP1-19 and *P. falciparum* MSP1-19, respectively. Interestingly, residents of ZG experienced high exposure to *P. vivax* (20%) but low exposure to *P. falciparum* (5%). Approximately 7% of the total seropositive participants had antibodies against both antigens (Table 2; Additional file 2: Fig. S2).

**Fig. 2:**
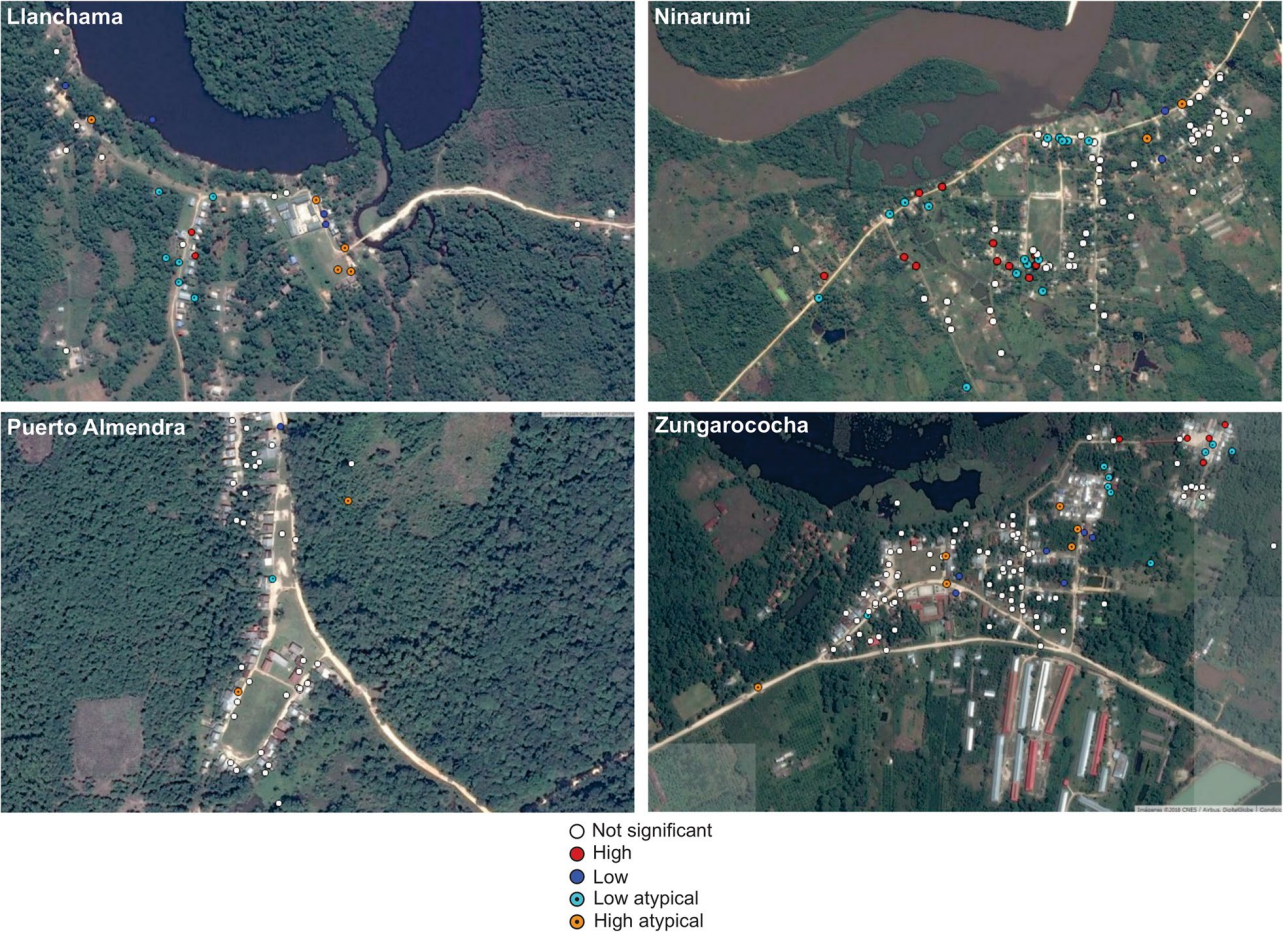
Spatial distribution of statiscally significant *Plasmodium*-infected households clusters identified in the four villages of the community of Zungarococha

The spatial distribution of infected households per site is shown in Fig. 2. Statistically significant spatial clusters of infected households were detected in the middle of the NR and LL villages. At ZG, was identified a cluster at the northeastern site, approximately 500 m from the village centroid. Multivariate adjusted risk factor analysis was conducted for any malaria infection. Only village and age remained independently associated with malaria infection. Individuals living in LL (AOR: 4.9, 95% CI [2.5–9.6]), PA (AOR 3.3, 95% CI [1.7–6.6]), and NR (AOR 2,6, 95% CI [1.5–4.6]) were more likely to be infected than those living in ZG. Additionally, individuals between 17 and 36 years of age had higher chances of malaria infection (AOR: 1.7, 95% CI [0.8–4.0]) compared to children aged under 15 years (Table 3).

**Table 3:** Univariate, multivariate, and backward selected model for malaria infection in the community of Zungarococha

Independent variable	Dependent variable		
	Malaria infection		
	OR (95% CI)	AOR (95% CI)^1^	AOR (95% CI)^2^
Sex			
Male	Ref	Ref	Ref
Female	0.8 (0.6–1.1)^b^	0.5 (0.3–1.0)^b^	0.5 (0.3–0.8)^c^
Age			
[15, 17]	Ref	Ref	Ref
(17,36]	1.7 (0.8–3.8)	1.7 (0.8–4.0)	1.7 (0.8–3.9)
(36,85]	1.1 (0.5–2.4)	0.9 (0.4–2.2)	0.9 (0.4–2.1)
Village			
Zungarococha	Ref	Ref	Ref
Llanchama	4.6 (2.4–8.7)^c^	2.9 (1.1–7.4)^b^	4.9 (2.5–9.6)^c^
Ninarumi	2.3 (1.3–4.0)^b^	1.7 (0.8–3.6)	2.6 (1.5–4.6)^c^
Puerto Almendra	3.0 (1.5–5.9)^c^	2.2 (1.0–5.0)^a^	3.3 (1.7–6.6)^c^
Occupation			
Other	Ref	Ref	
Outdoor^3^	2.0 (1.2–3.4)^b^	1.1 (0.5–2.3)	
Electricity availability			
Yes	Ref	Ref	
No	2.3 (1.2–4.3)^b^	1.1 (0.5–2.4)	
Wall material			
Wood	Ref	Ref	
Concrete	0.4 (0.3–0.6)^c^	0.8 (0.4–1.4)	
Triplay	0.8 (0.1–3.4)	1.1 (0.2–4.7)	
Floor material			
Wood	Ref	Ref	
Concrete	0.2 (0.1–0.7)^b^	0.6 (0.2–2.1)	
Soil or Sand	0.6 (0.3–1.6)	0.9 (0.3–2.6)	
Bed net usage			
Always	Ref	Ref	
Never	1.2 (0.7–2.0)	1.3 (0.7–2.4)	
Events		101	
*P. vivax*		96	
*P. falciparum*		10	
Observations		517	
Akaike Information Criterion		494.3	484.4

^1^ For all covariates

^2^ For covariates after backward elimination

^3^ Outdoor: includes Farmer, Guard, Logger, or Fisher

^a^*P* < 0.1; ^b^*P* < 0.05; ^c^*P* < 0.01

Considering *P. vivax* exposure, multivariate adjusted risk factor analysis showed that village, age, and bed net usage remained independently associated with *P. vivax* seropositivity. Individuals living in PA had higher odds ratios (AOR: 2.3, 95% CI [1.3–4.2]) than those living in LL, NR, and ZG. Furthermore, individuals aged between 36 and 85 years had the highest odds (AOR: 1.7, 95% CI [0.9–3.3]). Lastly, individuals who had never used bed nets also had higher odds of being exposed (AOR: 2.2, 95% CI [1.3–3.5]) (Table 4).

**Table 4:** Univariate, multivariate, and backward selected model for species-specific malaria exposure in the community of Zungarococha

Independent variable	Dependent variable					
	*P. vivax* exposure			*P. falciparum* exposure		
	OR (95% CI)	AOR (95% CI)^1^	AOR (95% CI)^2^	OR (95% CI)	AOR (95% CI)^1^	AOR (95% CI)^2^
Sex						
Male	Ref	Ref	Ref	Ref	Ref	Ref
Female	1.0 (0.7–1.5)	1.1 (0.7–1.7)	1.4 (1.0–2.2)	1.9 (1.0–3.6)^b^	1.9 (1.1–3.7)^b^	
Age						
[15, 17]	Ref	Ref	Ref	Ref	Ref	Ref
(17,36]	0.8 (0.4–1.6)	0.9 (0.5–1.8)	0.9 (0.5–1.8)	2.4 (1.1–6.0)	1.8 (0.7–4.7)	1.7 (0.7–4.5)
(36,85]	1.5 (0.8–2.9)	1.7 (0.9–3.4)	1.7 (0.9–3.3)	3.2 (1.5–7.9)^b^	2.7 (1.1–7.2)^b^	2.6 (1.1–6.8)^b^
Village						
Zungarococha	Ref	Ref	Ref	Ref	Ref	Ref
Llanchama	0.8 (0.4–1.5)	1.1 (0.4–2.7)	4.9 (0.4–1.7)	7.7 (3.9–15.4)^c^	5.6 (2.1–15.6)^c^	6.3 (2.7–15.4)^c^
Ninarumi	1.1 (0.7–1.7)	1.2 (0.6–2.3)	2.6 (0.6–1.6)	5.8 (3.3–10.2)^c^	5.8 (2.8–12.9)^c^	6.2 (3.0–13.0)^c^
Puerto Almendra	2.4 (1.4–4.3)^b^	2.5 (1.2 –5.2)^b^	3.3 (5.0–18.8)^c^	2.3 (1.3–4.2)^c^	12.2 (5.2–30.0)^c^	13.2 (6.0–30.0)^c^
Occupation						
Other	Ref	Ref		Ref	Ref	Ref
Outdoor^3^	1.0 (0.6–1.6)	1.0 (0.5–2.0)		1.4 (0.8–2.4)	1.7 (0.8–3.8)	1.8 (0.8–4.0)
Electricity availability						
Yes	Ref	Ref		Ref	Ref	
No	1.2 (0.6–2.2)	1.7 (0.7–3.7)		3.6 (1.9–6.6)^b^	1.4 (0.6–3.0)	
Wall material						
Wood	Ref	Ref		Ref	Ref	
Concrete	1.0 (0.7–1.5)	1.2 (0.7–2.1)		0.4 (0.2–0.5)^b^	(0.5–1.9)	
Triplay	0.7 (0.1–3.0)	0.9 (0.1–4.0)		2.7 (0.7–11.3)	4.6 (1.0–24.7)^a^	
Floor material						
Wood	Ref	Ref		Ref	Ref	Ref
Concrete	2.6 (0.9–11.5)	3.4 (0.9–16.6)^a^		(0.1–0.9)^c^	0.5 (0.2 -1.5)	0.5 (0.2 -1.4)
Soil or Sand	2.7 (0.9–11.7)	3.1 (0.9 -14.2)		0.4 (0.1–0.9)^b^	0.4 (0.1–1.0)^a^	0.4 (0.1–0.9)^b^
Bed net usage						
Always	Ref	Ref	Ref	Ref	Ref	Ref
Never	2.4 (1.5–3.8)^c^	2.3 (1.4–3.8)^c^	2.2 (1.3–3.5)^c^	0.7 (0.4–1.2)	0.6 (0.3–1.1)^a^	0.5 (0.3–1.0)^a^
Events		148			122	
Observations		517			517	
Akaike Inf. Crit		612.2	603.3		495.3	493.7

^1^ For all covariates

^2^ For covariates after backward elimination

^3^ Outdoor: includes Farmer, Guard, Logger, or Fisher

^a^*P* < 0.1; ^b^*P* < 0.05; ^c^*P* < 0.01

For *P. falciparum* exposure, sex, age, village, outdoor occupation, and bed net usage remained independent risk factors associated with *P. falciparum* seropositivity. Females had higher odds than males (AOR: 1.9, 95% CI [1.1–3.7]). Additionally, adults older than 36 years had higher odds ratios (AOR: 2.6, 95% CI [1.1–6.8]) compared to the rest of the population. Individuals living in PA had higher odds ratios (AOR: 13.2, 95% CI [6.0–30.0]) than those living in LL, NR, and ZG. People working outdoors were more likely to be exposed to *P. falciparum* (AOR: 1.8, 95% CI [0.8–4.0]). Finally, individuals who had never used bed nets also had higher odds of being exposed (AOR: 0.5 95% CI [0.3–1.0]). (Table 4).

**Fig. 3:**
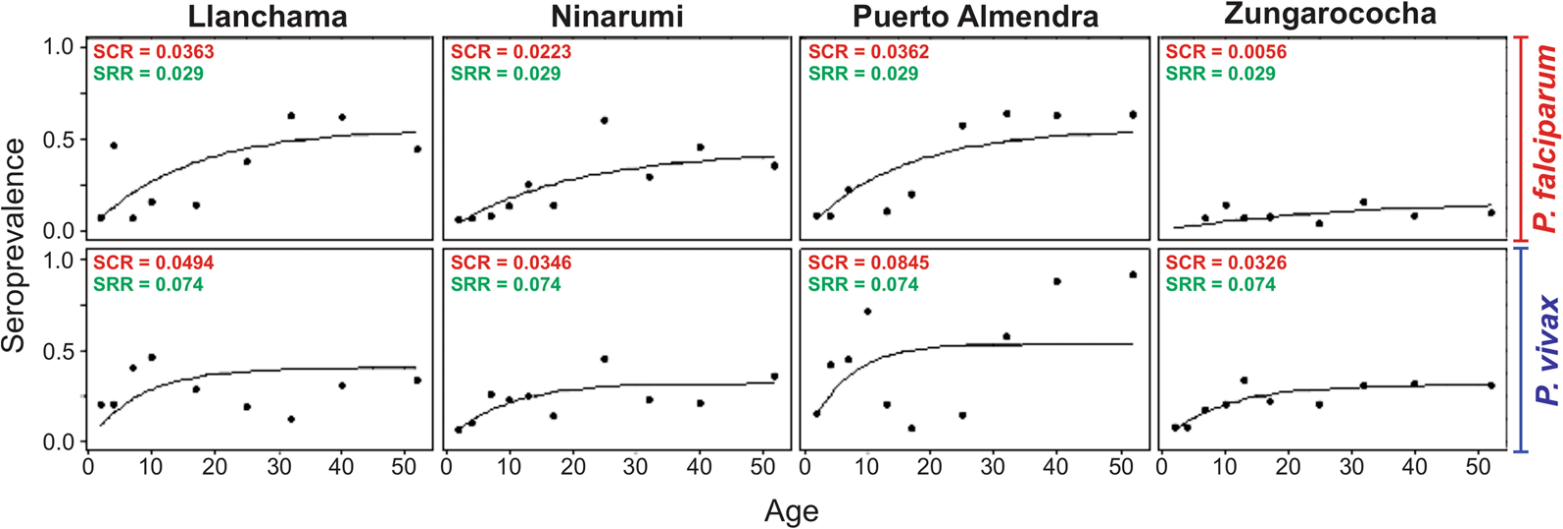
Age-stratified seroconversion rates for PfMSP1-119 kDa and PvMSP1-119 kDa per village, in the community of Zungarococha. Seropositive data were obtained using age deciles and fitted to reversible catalytic seroconversion models. Points show the observed values within each age group and the black line shows the fitted curve. The seroconversion (SCR) and seroreversion (SRR) rates are shown for each village and recombinant antigen

Seroprevalence curves showed different species- and community-specific patterns. Although *P. vivax* infections had a higher SCR than *P. falciparum* infections, these rates varied between villages. Assuming a constant malaria transmission intensity and a constant SRR per species between villages (*P. vivax*: ρ = 0.074, *P. falciparum*: ρ = 0.029), PA (*P. vivax*: λ = 0.0845, *P. falciparum*: λ = 0.0362) and LL (*P. vivax*: λ = 0.0494, *P. falciparum*: λ = 0.0363) had the highest SCR for both species. In contrast, ZG had the lowest SCR for both species (*P. vivax*: λ = 0.0326; *P. falciparum*: λ = 0.0056) (Fig. 3).

## Discussion

MINSA has implemented the Zero Malaria Plan initiative in the Loreto region to minimize or eliminate malaria cases in this region. Thanks to the progress made by this initiative, in 2022 the Peruvian government approved the second phase, the Plan towards Elimination of Malaria in Peru (2022–2030), which aims to eliminate malaria in all malaria-endemic regions of Peru (https://cdn.www.gob.pe/uploads/document/file/2793755/Norma_compressed.pdf.pdf?v=1643554873). To achieve malaria elimination, new diagnostic tools have been evaluated and used to monitor changes in transmission intensity, which can help improve the targeting of control interventions.

This cross-sectional study combined microscopical, molecular, and serological tools to detect current infections and past malaria exposure, making it possible to characterize *Plasmodium* transmission patterns in the endemic community of Zungarococha (Iquitos, Peru). In low transmission areas, the current metrics of malaria risk are affected by the different performance of current diagnostic tools to detect the presence of infected individuals with low parasite densities or without clinical symptoms. In such settings, serological methods have proven to be the most useful tools for identifying disease hotspots, as demonstrated in studies from Peru [2, 7], Brazil [27], and Vanuatu [6].

The survey was conducted between July and August 2015, during the peak incidence of clinical malaria cases. The prevalence of malaria determined using PCR data (18%) was three times higher than that determined using microscopy (6%), which is considered the gold standard. This discrepancy reflects the significant proportion of asymptomatic infections with low or absent parasite densities, primarily in low-endemicity settings, which can only be detected using highly sensitive PCR methods. Previous studies in rural Iquitos villages have also reported a high prevalence of asymptomatic parasite carriers (6–17%) during periods of high seasonal transmission, using PCR or quantitative real-time PCR methods [3, 28, 29].

The majority of malaria infections detected by PCR were submicroscopic and asymptomatic, which is commonly reported in Peruvian and Brazilian Amazonian communities, with *P. vivax* as the predominant species over *P. falciparum* [2, 3, 29–32]. As a result, asymptomatic individuals with chronic infections act as reservoirs that contribute to maintaining malaria transmission, posing a serious challenge for elimination efforts [33, 34]. As mentioned previously, increases in asymptomatic malaria, especially in remote rural areas, directly affect populations with economic activities conducted in those areas as well as national and foreign-deployed armed forces. Microscopical, molecular, and serological approaches indicated that species-specific malaria transmission varied widely across villages. LL and PA had the highest prevalence of *P. vivax* according to microscopy (11% and 6%, respectively) and PCR (27% and 25%, respectively). These two communities also showed the highest seroprevalence and seroconversion rate (SCR) estimates for both *Plasmodium* species*.*

Seroepidemiological studies have been reported as useful tools to measure transmission intensity when parasite prevalence is low or scarce [6, 8, 9] because many of the asymptomatic infections identified are probably due to previous exposure with suppressed parasite densities at submicroscopic levels [35]. Since the 1970s, serology has been a useful tool for measuring exposure to malaria and has been reported as a prominent method in early elimination attempts. With advances in technology, serology has become an effective tool for measuring malaria transmission [5, 36, 37]. Although, as promising as serology, there is still a need to standardize the ELISA protocols and select the best antigens, which may depend on the area under study [19, 27, 38].

The analyses of prevalence and seroprevalence were different in the four villages, with species- and community-specific transmission patterns, demonstrating a high degree of heterogeneity in the transmission of both *Plasmodium* species within these geographically close communities, separated by only 2 km. *P. vivax* MSP1-19 seroprevalence was higher than *P. falciparum* MSP1-19, and only 6% of the total seropositive participants had antibodies against both antigens. Moreover, the lower *P. falciparum* seroprevalence observed in ZG confirms the predominance of *P. vivax* over *P. falciparum* in this region, corroborating previous studies [2, 10] and in this study area, the Zungarococha community [3, 28], have also reported a high prevalence of asymptomatic parasite carriers, detected only by PCR, with a predominance of *P. vivax* over *P. falciparum*. It should be noted that the differences in the specific seroprevalence of the species between villages are consistent with previous entomological results, which indicate a high heterogeneity in malaria transmission in the peri-Iquitos region [39, 40], even though the vector *Anopheles darlingi* populations are highly homogeneous [41]. The levels of IgG antibodies observed for the MSP1-19 protein for both species were robust and sensitive, including in PA, where no prevalence cases of *P. falciparum* were found by PCR, demonstrating the presence of low levels of parasite exposure in these communities, located 5–10 km from Iquitos [3], where the malaria transmission rate is less than 0.5 infection/person/year [3, 11]. Serological markers can be used to determine exposure and risk factors for malaria.

The multivariate adjusted risk factor analysis, age, village, and limited bed net usage were associated with *P. vivax* seropositivity, while sex, village, age, outdoor occupation, and limited bed net usage were associated with *P. falciparum* seropositivity. It has been reported that some ecological factors (secondary forest and natural water bodies) or human socioeconomic activities (outdoor activities) may play an important role in increasing exposure to mosquito bites in the study area [2, 42, 43], just as the effect of age on seroprevalence usually reflects the cumulative exposure to malaria infections occurring with age rather than a higher risk of exposure in adults than in children [7, 8, 44]. This limited bed net usage was a risk factor for malaria, confirming the importance of using it as a preventive measure against malaria in these villages.

The catalytic model used to generate the seroprevalence curves showed different species- and village-specific patterns, suggesting that at the age of 20 years, a constant rate of exposure is reached that will maintain seropositivity (close to or greater than 40% depending on the species and village). The present analysis attempted to obtain estimates of SCR and SRR, knowing that both could vary by the exposure history of the study population and that the probability of SRR can occur at any time because it is independent of how long the individual has been positive [45]. LL and PA showed the highest SCR estimates for both species, with a very high seroprevalence, mainly in PA, suggesting a recent increase in infections for both species in this village. The SCR rates for the PvMSP1-19 and PfMSP1-19 antigens were highly correlated with parasite exposure, providing information on current and recent infections.

Several studies have demonstrated the effectiveness of seroprevalence in distinguishing sites with varying levels of endemicity in relation to the parasite incidence [46, 47]. MSP1-19 was selected for further analysis due to its ability to elicit a more robust antibody response. Even one reported prior infection was sufficient to produce a positive anti-MSP1-19 IgG response for more than 5 months in the absence of reinfection at the study site [11], corroborating the claim that it is an optimal marker for malaria exposure [48], which is species-specific [49] despite the considerable homology in the gene sequences of MSP1-19 antigens of *Plasmodium* species [50]. Nonetheless, it is recommended to use multiple antigens for each species because variability in immunogenicity cannot be excluded in order to optimize ELISA sensitivity and to improve the identification of malaria transmission dynamics in low transmission areas [6, 7, 9, 38].

These seroprevalence curves are similar to those previously described by other authors [2], who suggested that there is insufficient data to establish their role in determining the heterogeneity of malaria transmission in the Peruvian Amazon. Although it is known that malaria infection leaves an “antibody footprint” that will last longer than the infection itself, cumulative malaria exposure in a population can be considered as an alternative tool to measure the intensity of malaria transmission as well as to assess changes in exposure [46] which may lead to the success of interventions in a given area [6]. Other authors recommend that both serological data in conjunction with mathematical modelling used for data analysis provide a powerful approach to inform epidemiologists on malaria transmission intensity and its putative changes over time [19].

The cross-sectional survey was conducted in July 2015 in a remote rural area of Loreto, prior to the implementation of the Malaria Zero Program. The resulting data provide a valuable baseline resource for measuring the program's impact on malaria reduction and the implementation of eradication campaigns. Furthermore, the epidemiology of malaria at the time of study execution can be compared to that conducted in urban and rural settings as part of more recent interventions against malaria.

## Conclusions

The diversity of malaria transmission in the malaria-endemic zones of the Peruvian Amazon Basin is striking. As the transmission rates decline, particularly in residual malaria scenarios, this heterogeneity becomes even more pronounced. The combination of molecular and serological techniques significantly enhances the ability to identify both current infections and past exposure to malaria. Furthermore, this approach helps to identify epidemiological risk factors in the malaria-endemic areas of the Loreto region. Such advancements are crucial, especially in regions with low transmission rates that are either currently implementing or planning elimination programs, such as the Malaria Zero Program in the Peruvian Amazon.

### Supplementary Information

**Additional file 1: Fig. S1.** Age-stratified malaria infection proportions by Plasmodium species and diagnosis method in four villages of the community of Zungarococha

**Additional file 2: Fig. S2.** Age-stratified antibody (IgG) levels against the recombinant antigens PfMSP1-119 kDa and PvMSP1-119 kDa in the four villages of the community of Zungarococha, Loreto, Peru. Vertical lines show the cut-off values for each recombinant antigen defined by the mixture models.

## Data Availability

All necessary data supporting the findings of this study have been comprehensively included in the article. Additional information may be acquired from the corresponding author upon request, and subject to reasonable conditions.

## Abbreviations

AIC: Akaike information criterion
ELISA: Enzyme-linked immunosorbent assay
IgG: Immunoglobulin G
OR: Odds ratio
PCR: Polymerase chain reaction
SCR: Seroconversion rate
SSU rRNA: Small subunit ribosomal RNA
SRR: Seroreversion rate
