# Supplementary figures and images for: Unravelling heterogeneous malaria transmission dynamics in the Peruvian Amazon: insights from a cross-sectional survey
Source: Malar J. 2024 Jul 15;23:209. doi: 10.1186/s12936-024-05032-8 (PMC11251108; doi:10.1186/s12936-024-05032-8)

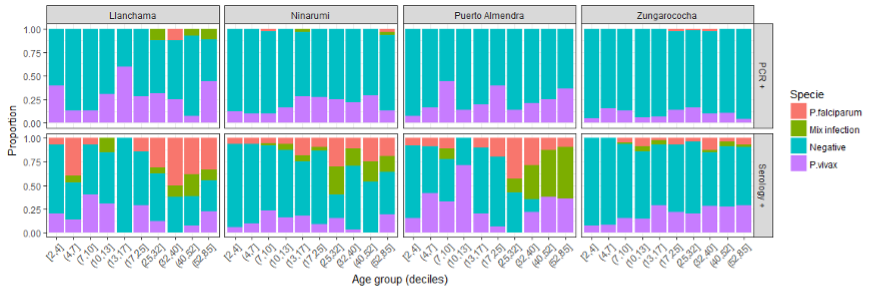

Supplement: Supplementary file 1 — Additional file 1: Fig. S1. Age-stratified malaria infection proportions by Plasmodium species and diagnosis method in four villages of the community of Zungarococha [file 12936_2024_5032_MOESM1_ESM.png]

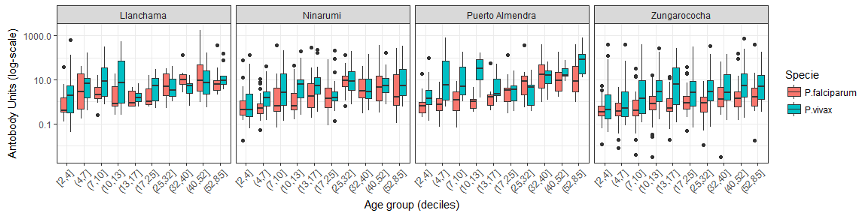

Supplement: Supplementary file 2 — Additional file 2: Fig. S2. Age-stratified antibody (IgG) levels against the recombinant antigens PfMSP1-119 kDa and PvMSP1-119 kDa in the four villages of the community of Zungarococha, Loreto, Peru. Vertical lines show the cut-off values for each recombinant antigen defined by the mixture models. [file 12936_2024_5032_MOESM2_ESM.png]
